# Supplementary material for: Black-boxing and cause-effect power
Source: PLoS Comput Biol. 2018 Apr 23;14(4):e1006114. doi: 10.1371/journal.pcbi.1006114 (PMC5933815; doi:10.1371/journal.pcbi.1006114)
Supplement: S1 Text — Detailed cause-effect structures for the examples presented in the main text. (DOCX) [file pcbi.1006114.s001.docx]

**Black-boxing and cause-effect power:**

**Supplementary Information**

William Marshall^1^, Larissa Albantakis^1^, Giulio Tononi^1,^ *^*^*

^1^*Department of Psychiatry, Center for Sleep and Consciousness, University of Wisconsin, Madison, WI, USA*

*^*^Corresponding author: gtononi@wisc.edu*

S1 Text – Full analysis of cause-effect power

Here we present a more detailed account of the integrated information analysis of the example systems discussed in the main text. All calculations were performed using the PyPhi software package in Python (Mayner et al., 2016).

For a given system in a specific state, the first step involves identifying its cause-effect structure, the set of mechanisms in the system. A mechanism is a set of elements that irreducibly constrains the past and future states of the system. Each member of the power set of system elements is tested as a potential mechanism. The set of system elements whose past states are most irreducibly constrained by the mechanism are its past purview (evaluated by the cause integrated information of the mechanism ϕ_cause_). The set of system elements whose future states are most irreducibly constrained by the mechanism are the mechanism’s future purview (evaluated by the effect integrated information ϕ_effect_). The way that a mechanism, by being in its current state, constrains its purview elements is captured by its cause-effect repertoire, a pair of probability distributions over the past and future states of the purview elements (e.g. Fig. 3, main text). Note that these probabilities are obtained from the system’s transition probability matrix (TPM) assuming a maximum entropy distribution for the marginal distribution of all possible past states. This corresponds to setting the system into all possible states with equal likelihood performing an interventionist causal analysis. ϕ_cause_ and ϕ_effect_ quantify the difference between the cause-effect repertoire, and the cause-effect repertoire under a partition of the mechanism as the earth-mover’s distance between the two probability distributions (Oizumi et al., 2014). The overall integrated information of a mechanism is then the minimum of its ϕ_cause_ and ϕ_effect_. In sum, the complete specification of a mechanism thus includes its cause and effect purviews (the elements over which it has maximally irreducible power to constrain the past and future states), the cause-effect repertoires that specify those constraints, and its integrated information value (ϕ). The set of all mechanisms constitutes the system’s cause-effect structure.

For a micro-level system, mechanisms are composed of micro elements and cannot include macro elements. Conversely, if a macro-level system is analyzed, only compositions of macro elements are tested and the potential causes and effects of individual micro elements within the black boxes are ignored. In other words, each level has a particular TPM, obtained from perturbing the system into all possible states with equal likelihood at that micro or macro level, which then determines the system’s mechanisms at this particular level.

Next, to obtain the integrated information of the system, all possible directed partitions of the system are considered, to find the one that least affects the system’s cause-effect structure. After each partition, the cause-effect structure is recalculated, and the result is compared to the cause-effect structure of the whole system. The partition that makes the least difference to the cause-effect structure is the minimum information partition (MIP), and the difference it makes, as measured using an extended earth movers distance (Oizumi et al., 2014), defines the integrated information of the system (Φ). Note that, for Φ, i.e. integrated information at the system level, all possible directed partitions of constituent micro elements are evaluated, regardless of whether the system is defined at a micro or macro level. This excludes the possibility to ‘hide’ micro elements without cause-effect power inside a black box, which would trivially increase the system’s integration. In sum, the cause-effect structure (the set of all mechanisms) of a macro level system is evaluated purely at the macro level; its irreducibility, however, is evaluated by the partition between micro elements that makes the least difference to the macro cause-effect structure.

**
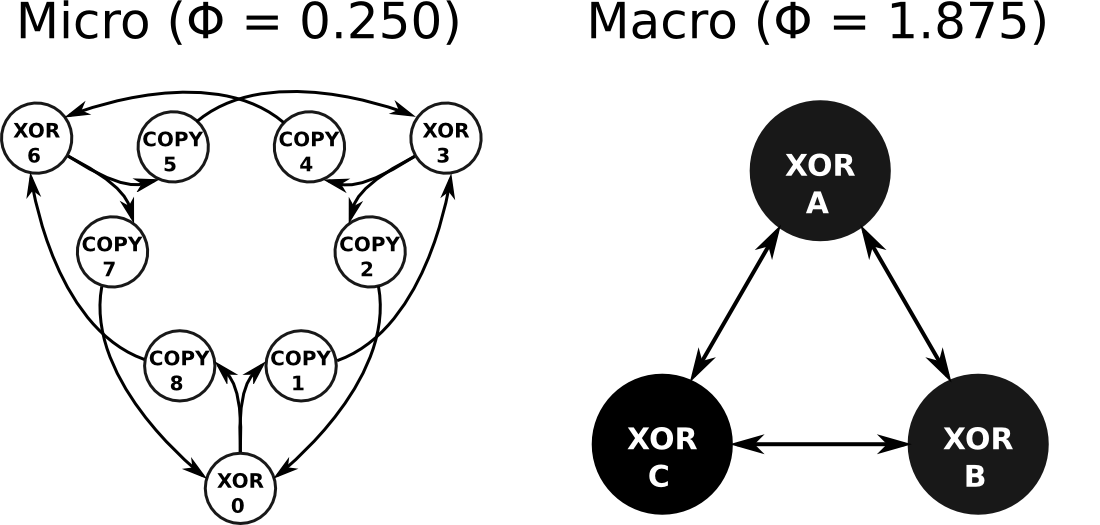
**

**Figure A**: Systems of micro elements with labels to facilitate description of the mechanisms. Left: Micro system with elements labeled 0-8. Right: Macro system with elements labeled A-C. All elements are in the ‘OFF’ state.

Example 1 – Composition and Integration

To describe the cause-effect structure of the micro system, we first assign labels to each of the micro elements in the system, as shown in Fig. A. There are three mechanisms in the cause-effect structure, they are all first order mechanisms and each one corresponds to an element implementing XOR logic.

| 0-8  unpartitioned | Mechanism | Past Purview | Future Purview | ϕ |
| --- | --- | --- | --- | --- |
|  | (0) | (2, 7) | (1, 8) | 0.5 |
|  | (3) | (1, 5) | (2, 4) | 0.5 |
|  | (6) | (4, 8) | (5, 7) | 0.5 |

To assess the integrated information Φ of the micro system, we identify the unidirectional system partition that makes the least difference to the cause-effect structure, termed the minimum information partition (MIP). For this system, the MIP is to cut all connections from (0, 1, 2, 3, 4, 5, 6, 7) to (8). Under this partition, the mechanism specified by element 0 is altered, its future purview is reduced from (1, 6) to only (1). Note that numbers in bold refer to mechanisms for which the partitioned cause-effect structure which is different from the unpartitioned cause-effect structure. Assessing the difference between the unpartitioned and partitioned cause-effect structure of the micro system using an extended earth-mover’s distance (Oizumi et al., 2014), the resulting integrated information value of the micro system is Φ = 0.25.

| 0-8  partitioned | Mechanism | Past Purview | Future Purview | ϕ |
| --- | --- | --- | --- | --- |
|  | **(0)** | **(2, 7)** | **(1)** | **0.5** |
|  | (3) | (1, 5) | (2, 4) | 0.5 |
|  | (6) | (4, 8) | (5, 7) | 0.5 |

Next, we describe the cause-effect structure of the macro system displayed in Fig. A. The three black-box macro elements are constituted of micro elements A = (0, 2, 7), B = (1, 3, 4) and C = (5, 6, 8) with corresponding output elements (0), (4) and (6). This black-box system has three high-order mechanisms.

| ABC  unpartitioned | Mechanism | Past Purview | Future Purview | ϕ |
| --- | --- | --- | --- | --- |
|  | (A, B) | (A, B, C) | (C) | 0.5 |
|  | (A, C) | (A, B, C) | (B) | 0.5 |
|  | (B, C) | (A, B, C) | (A) | 0.5 |

The MIP for this macro system cuts connections from (1, 3) to (0, 2, 4, 5, 6, 7, 8). After the partition, all of the mechanisms have been destroyed. Mechanisms (A, B) and (B, C) no longer have irreducible causes or effects, while the set of elements (A, C) has an effect but no cause. The integrated information of this system is Φ = 1.875. Note that the MIP is a partition of micro elements; yet the black-box system has higher Φ because the partition affects the macro cause-effect structure more than it would affect the cause-effect structure of the corresponding micro system. This is because the mechanisms at the macro level are high-order mechanisms that constrain larger parts of the system (have larger purviews). These macro constraints are completely lost even under the micro partition that makes the least difference.

| ABC  partitioned | Mechanism | Past Purview | Future Purview | ϕ |
| --- | --- | --- | --- | --- |
|  | **(A, B)** | **()** | **()** | **0** |
|  | **(A, C)** | **()** | **(B)** | **0** |
|  | **(B, C)** | **()** | **()** | **0** |

Example 2 – Local Maxima

In this example with 55 elements we will not assign numbers to the elements. Instead, we will simply refer to each element based on the number of inputs and outputs it has, e.g., NOR(3, 1) for a NOR element with three inputs and one output. Each of the 55 elements specifies a first order mechanism, summarized in the table below.

| unpartitioned – micro | | | | |
| --- | --- | --- | --- | --- |
| Multiplicity | Mechanism | Past Purview | Future Purview | ϕ |
| 30 | NOR(1, 1) | NOR(1, 6) | NOR(2, 1) | 0.25 |
| 15 | NOR(2, 1) | 2*NOR(1, 1) | NOR(3, 1) | 0.125 |
| 5 | NOR(3, 1) | 3*NOR(2, 1) | NOR(1, 6) | 0.5 |
| 5 | NOR(1, 6) | NOR(3, 1) | 6*NOR(1, 1) | 0.25 |

The minimum information partition (MIP) for this system is to cut the connections from a NOR(1, 1) element to the rest of the system. It doesn’t matter which NOR(1, 1) element as they all have the same effect on their respective future purview. The result of the MIP is that one mechanism is destroyed, and another is altered. The integrated information of this system is Φ = 0.453.

| partitioned – micro | | | | |
| --- | --- | --- | --- | --- |
| Multiplicity | Mechanism | Past Purview | Future Purview | ϕ |
| 29 | NOR(1, 1) | NOR(1, 6) | NOR(2, 1) | 0.25 |
| **1** | **NOR(1, 1)** | **NOR(1, 6)** | **()** | **0** |
| 14 | NOR(2, 1) | 2*NOR(1, 1) | NOR(3, 1) | 0.125 |
| **1** | **NOR(2, 1)** | **NOR(1, 1)** | **NOR(3, 1)** | **0.125** |
| 5 | NOR(3, 1) | 3*NOR(2, 1) | NOR(1, 6) | 0.5 |
| 5 | NOR(1, 6) | NOR(3, 1) | 6*NOR(1, 1) | 0.25 |

One option for a macro system is to define black-box elements that implement AND and OR logic (Fig. 5 and 6, main text). This system has an average spatial grain size of 2.75. There is a symmetry in the system, so that the mechanisms specified by each OR gate are the same, and the mechanisms specified by each AND gate are also the same (the OR elements output to six AND elements and take inputs from three AND elements, while the AND elements all take inputs from two OR elements and output to one OR element). At this macro scale, the system has 15 black-box elements implementing AND logic and 5 black-box elements implementing OR logic, over two time steps, and each specifies a first order mechanism.

| unpartitioned – AND/OR black boxes | | | | |
| --- | --- | --- | --- | --- |
| Multiplicity | Mechanism | Past Purview | Future Purview | ϕ |
| 5 | OR | 3*AND | 6*AND | 0.071 |
| 15 | AND | 2*OR | OR | 0.125 |

The MIP for this system is to cut the outputs of a NOR(2, 1) element that is one of the input elements of an AND black-box element. In this case, the mechanisms have the same cause-effect power, but one of the AND mechanisms and one of the OR mechanisms have reduced purviews, constraining less elements.

| partitioned – AND/OR black boxes | | | | |
| --- | --- | --- | --- | --- |
| Multiplicity | Mechanism | Past Purview | Future Purview | ϕ |
| 4 | OR | 3*AND | 6*AND | 0.071 |
| **1** | **OR** | **3*AND** | **5*AND** | **0.071** |
| 14 | AND | 2*OR | OR | 0.125 |
| **1** | **AND** | **OR** | **OR** | **0.125** |

Another black-box system at a coarser macro scale has five black-box elements {A, B, C, D, E} over 4 time steps. Each black box implements a MAJORITY function over its three inputs, with a specialized connectivity pattern shown in Fig. 6, main text. Of the 31 (2^N^-1) possible mechanisms from the power set of 5 elements, 30 specify irreducible past and future constraints:

| unpartitioned – MAJORITY black box | | | |
| --- | --- | --- | --- |
| Mechanism | Past Purview | Future Purview | ϕ |
| (A) | (C, D, E) | (B, C, D) | 0.25 |
| (B) | (A, D, E) | (C, D, E) | 0.25 |
| (C) | (A, B, E) | (A, D, E) | 0.25 |
| (D) | (A, B, C) | (A, B, E) | 0.25 |
| (E) | (B, C, D) | (A, B, C) | 0.25 |
| (A, B) | (A, C, E) | (C, D) | 0.2 |
| (A, C) | (A, B, C, D, E) | (A, B, C, D, E) | 0.2 |
| (A, D) | (A, B, C, D, E) | (A, B, C, D, E) | 0.2 |
| (A, E) | (B, D, E) | (B, C) | 0.2 |
| (B, C) | (B, D, E) | (D, E) | 0.2 |
| (B, D) | (A, B, C, D, E) | (A, B, C, D, E) | 0.2 |
| (B, E) | (A, B, C, D, E) | (A, B, C, D, E) | 0.2 |
| (C, D) | (B, C, E) | (A, E) | 0.2 |
| (C, E) | (A, B, C, D, E) | (A, B, C, D, E) | 0.2 |
| (D, E) | (A, C, D) | (A, B) | 0.2 |
| (A, B, C) | (A, B, C, D) | (A, B, C, D, E) | 0.2 |
| (A, B, D) | (A, C, E) | (B, C, D, E) | 0.257143 |
| (A, B, E) | (A, B, C, E) | (A, B, C, D, E) | 0.2 |
| (A, C, D) | (B, C, E) | (A, B, D, E) | 0.257143 |
| (A, C, E) | (B, D, E) | (A, B, C, D) | 0.257143 |
| (A, D, E) | (A, B, C, D) | (A, B, C, D, E) | 0.2 |
| (B, C, D) | (B, C, D, E) | (A, B, C, D, E) | 0.2 |
| (B, C, E) | (B, D, E) | (A, C, D, E) | 0.257143 |
| (B, D, E) | (A, C, D) | (A, B, C, E) | 0.257143 |
| (C, D, E) | (A, C, D, E) | (A, B, C, D, E) | 0.2 |
| (A, B, C, D) | (A, B, C, D, E) | (A, B, C, D, E) | 0.185709 |
| (A, B, C, E) | (A, B, C, D, E) | (A, B, C, D, E) | 0.185709 |
| (A, B, D, E) | (A, B, C, D, E) | (A, B, C, D, E) | 0.185709 |
| (A, C, D, E) | (A, B, C, D, E) | (A, B, C, D, E) | 0.185709 |
| (B, C, D, E) | (A, B, C, D, E) | (A, B, C, D, E) | 0.185709 |

The minimum information partition of this network is to cut the outputs of one of the NOR(1, 1) micro elements. By the symmetry in the system, there is an equivalent MIP in each of the black-box elements; however, due to the specialized connectivity structure, not all NOR(1, 1) elements are equivalent. One MIP option is to cut the hidden NOR(1, 1) micro element in black-box element A that receives input from D and outputs to the NOR(2, 1) micro elements along with the NOR(1, 1) micro element that receives input from C. As a result of the MIP, two of the mechanisms are destroyed (BCD and ABCD) and 15 others are modified (shown in bold), resulting in a Φ value of 2.333.

| partitioned – MAJORITY black box | | | |
| --- | --- | --- | --- |
| Mechanism | Past Purview | Future Purview | ϕ |
| **(A)** | **(C, E)** | **(B, C, D)** | **0.25** |
| (B) | (A, D, E) | (C, D, E) | 0.25 |
| (C) | (A, B, E) | (A, D, E) | 0.25 |
| **(D)** | **(A, B, C)** | **(B, E)** | **0.25** |
| (E) | (B, C, D) | (A, B, C) | 0.25 |
| **(A, B)** | **(D, E)** | **(C, D)** | **0.227273** |
| **(A, C)** | **(A, B, C, E)** | **(A, B, C, D, E)** | **0.2** |
| **(A, D)** | **(A, B, C, E)** | **(B, C, D, E)** | **0.2** |
| **(A, E)** | **(B, C, E)** | **(B, C)** | **0.2** |
| (B, C) | (B, D, E) | (D, E) | 0.2 |
| **(B, D)** | **(A, B, C, D, E)** | **(B, C, D, E)** | **0.2** |
| (B, E) | (A, B, C, D, E) | (A, B, C, D, E) | 0.2 |
| **(C, D)** | **(B, C, E)** | **(A, B, D, E)** | **0.2** |
| (C, E) | (A, B, C, D, E) | (A, B, C, D, E) | 0.2 |
| (D, E) | (A, C, D) | (A, B) | 0.2 |
| **(A, B, C)** | **(A, B, C, D)** | **(A, B, C, D, E)** | **0.181816** |
| (A, B, D) | (A, C, E) | (B, C, D, E) | 0.257143 |
| (A, B, E) | (A, B, C, E) | (A, B, C, D, E) | 0.2 |
| **(A, C, D)** | **(B, C, E)** | **(B, D, E)** | **0.257143** |
| **(A, C, E)** | **(B, C, E)** | **(A, B, C, D)** | **0.257143** |
| **(A, D, E)** | **(A, B, C, D, E)** | **(A, B, C, D, E)** | **0.142158** |
| (B, C, E) | (B, D, E) | (A, C, D, E) | 0.257143 |
| (B, D, E) | (A, C, D) | (A, B, C, E) | 0.257143 |
| (C, D, E) | (A, C, D, E) | (A, B, C, D, E) | 0.2 |
| **(A, B, C, E)** | **(A, B, C, D, E)** | **(A, B, C, D, E)** | **0.207691** |
| **(A, B, D, E)** | **(A, B, C, D, E)** | **(A, B, C, D, E)** | **0.25** |
| **(A, C, D, E)** | **(A, B, C, D, E)** | **(A, B, C, D, E)** | **0.161903** |
| (B, C, D, E) | (A, B, C, D, E) | (A, B, C, D, E) | 0.185709 |
