# Supplementary material for: Black-boxing and cause-effect power
Source: PLoS Comput Biol. 2018 Apr 23;14(4):e1006114. doi: 10.1371/journal.pcbi.1006114 (PMC5933815; doi:10.1371/journal.pcbi.1006114)
Supplement: S2 Text — Additional examples exploring the effects of degeneracy and indeterminism. (DOCX) [file pcbi.1006114.s002.docx]

**Black-boxing and cause-effect power:**

**Supplementary Information**

William Marshall^1^, Larissa Albantakis^1^, Giulio Tononi^1,^ *^*^*

^1^*Department of Psychiatry, Center for Sleep and Consciousness, University of Wisconsin, Madison, WI, USA*

*^*^Corresponding author: gtononi@wisc.edu*

S2 Text – Black-boxing of degenerate or indeterministic systems

As demonstrated in (Hoel et al., 2013, 2016), the main factor enabling an increase in intrinsic cause-effect power through coarse-graining is a reduction in indeterminism and degeneracy at the macro level. An increase in intrinsic cause-effect power by reducing degeneracy is also possible through black-boxing, as shown below (“Degeneracy example”). However, black-boxing may increase indeterminism (see below “Propagation delay through noisy channel”).

Propagation delay through noisy channels

In Example 1 (main text), we demonstrated that black-boxing a system with deterministic propagation delay may lead to an increase in intrinsic cause-effect power, as it can increase system integration through emergent high-order mechanisms. However, indeterminism in the system may influence whether a black-boxed macro level can “beat” the micro level in terms of cause-effect power.

Here we explore the effect of noise on black-boxing by considering a scenario in which propagation delays occur over noisy channels. In this case, the COPY elements take a single input and then output the same value with probability *p* in [0.5, 1]. The original results of Fig. 2 (main text) refer to a noiseless channel (*p* = 1). A completely noisy channel (*p*= 0.5) would have no cause-effect power at any level (macro or micro). The integrated information analysis is performed on both the micro and black-box physical system for several different values of *p* (see Table 1). The number and orders of mechanisms is the same for all values of *p*> 0.5, but the ϕ value of each mechanism decreases as *p* decreases, and it does so more steeply for the black-box system than for the micro system. Along with ϕ, the overall integrated information Φ of both the black-box and micro systems decrease as the amount of noise increases. Since Φ of the black-box system declines faster with increasing noise than Φ of the micro system, the macro system “beats” the micro for *p* > 0.6, whereas the micro level system “wins” when *p* ≤ 0.6. The reduction in cause-effect power due to indeterminism can outweigh the increase due to high-order mechanisms, since indeterminism (noise) disproportionately affects high-order mechanisms. Therefore, several interrelated aspects of cause-effect power have to be considered when assessing if the “macro beats the micro,” including the presence of high-order mechanisms, indeterminism, and degeneracy.

**Table 1:** Integrated information for various noise levels (p) in the propagation delay network (Fig. 3 main text)

| *p* | 1.0 | 0.9 | 0.8 | 0.7 | 0.6 | 0.5 |
| --- | --- | --- | --- | --- | --- | --- |
| Micro Φ | 0.250 | 0.160 | 0.090 | 0.040 | 0.010 | 0.000 |
| Micro ϕ | 0.5 | 0.4 | 0.3 | 0.2 | 0.1 | 0 |
| Black box Φ | 1.875 | 1.046 | 0.363 | 0.070 | 0.004 | 0.000 |
| Black box ϕ | 0.500 | 0.320 | 0.180 | 0.080 | 0.020 | 0.000 |

Degeneracy example

Reducing degeneracy, the convergence of multiple past system states onto the same current system state, is one way in which a coarse-grained macro level can achieve higher cause-effect power than its corresponding micro level (Hoel et al., 2013, 2016). Here we show that black-boxing micro elements into macro elements can also be exploited to counteract degeneracy at the micro level.

Fig. A shows a system of six micro elements – four COPY gates and two AND gates that are black-boxed into two macro elements, which correspond to macro COPY gates. As illustrated by the TPM in Fig. A (left, bottom), the micro level has a high degree of degeneracy (*i.e.* multiple rows leading to the same column in the TPM). In the example, two COPY micro elements input to a single AND micro element, whose current state is OFF. This implies degeneracy in the system since three states of the COPY elements (OFF, OFF), (ON, OFF) and (OFF, ON) all lead to the same state of the AND element (OFF). The micro system in state ‘all OFF’ specifies six first order mechanisms; the AND elements specify mechanisms with ϕ = 0.167; and the COPY elements specify mechanisms with ϕ = 0.25. There are no high-order mechanisms. The integrated information for the micro system is Φ = 0.215.

We then consider the system at the macro level, after black-boxing each AND micro element with its two inputting COPY micro elements. Each black-box macro element turns out to implement, over two time steps, a macro COPY logic, specifying a first-order mechanism with ϕ = 0.5. Again, there are no high-order mechanisms. However, the macro system has no degeneracy, since no two past states converge onto the same current state (see macro TPM in Fig. A). For this reason, the integrated information of a COPY element in the macro system is higher (ϕ = 0.5) than in the micro system (ϕ = 0.25), and so is the overall integrated information for the black-box macro system (Φ = 0.639). Thus, appropriately black-boxing micro into macro elements reduces degeneracy and in doing so increases the cause-effect power of the system, as measured by integrated information Φ.


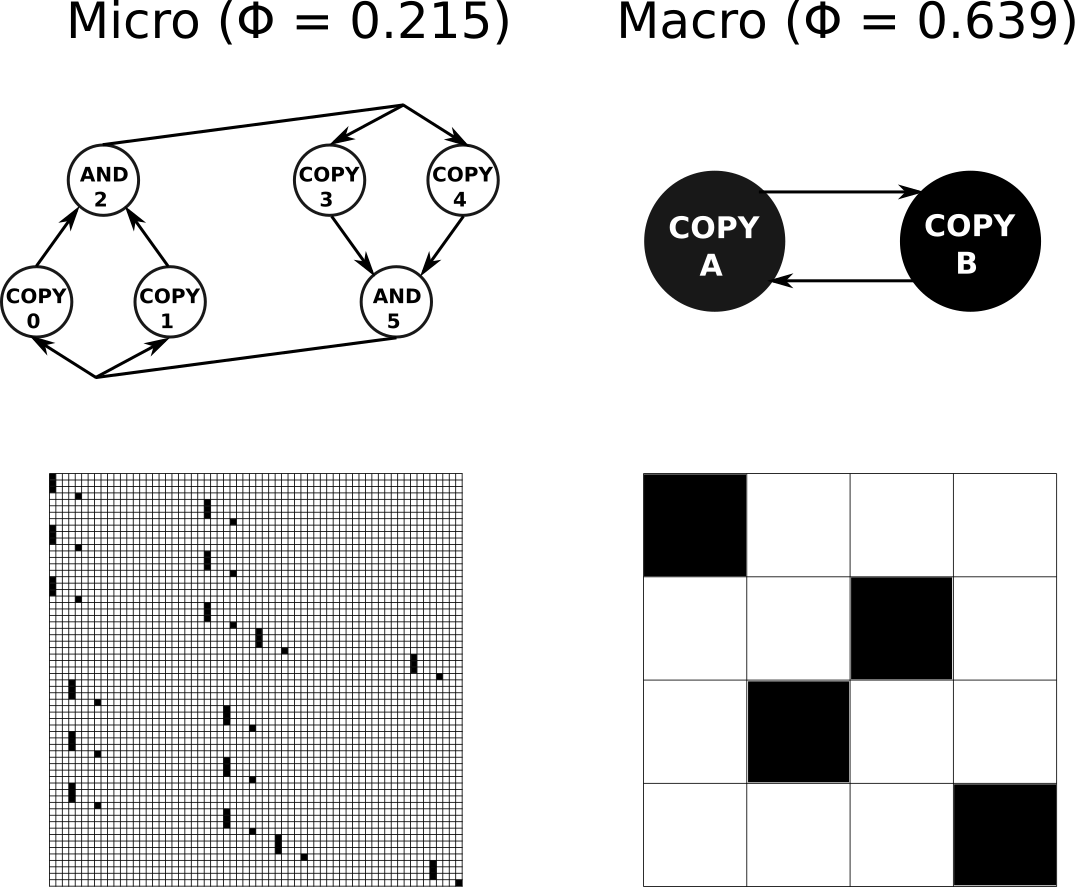


***Figure A:*** *Left: Two AND elements that each receive inputs from two COPY elements and send output to two other COPY elements, along with the TPM calculated from systematic perturbation of the elements. The current state of all micro elements is OFF. The integrated information of the micro system is Φ = 0.215. Right: Black-box elements consisting of AND elements as outputs, and the corresponding COPY elements hidden within. The current macro state of the black-box elements is OFF, corresponding to the current micro state of the micro elements that define their output. The TPM for this system is found by perturbing the inputs to the black-box element in all possible ways, it is determined that it implements COPY logic over two time steps. The black-box macro system has Φ = 0.639.*

To describe the cause-effect structure of the micro system in detail, we use the labels shown in Fig. A.

| micro  unpartitioned | Mechanism | Past Purview | Future Purview | ϕ |
| --- | --- | --- | --- | --- |
|  | (0) | (5) | (2) | 0.25 |
|  | (1) | (5) | (2) | 0.25 |
|  | (2) | (0, 1) | (3, 4) | 0.167 |
|  | (3) | (2) | (5) | 0.25 |
|  | (4) | (2) | (5) | 0.25 |
|  | (5) | (3, 4) | (0, 1) | 0.167 |

For this system, the MIP is to cut all connections from (1) to (0, 2, 3, 4, 5). Note that there are other equivalent MIPs; we focus on this specific cut without loss of generality. Under the partition, element 1 no longer has an effect and thus does not specify a mechanism, and the mechanism specified by element 2 is altered, as its past purview is reduced from (0, 1) to only (0). Comparing the unpartitioned and partitioned cause-effect structure, we find that the resulting integrated information value for the micro system is Φ = 0.215. Entries in bold highlight mechanisms in the partitioned cause-effect structure that are different from those in the unpartitioned cause-effect structure.

| micro  partitioned | Mechanism | Past Purview | Future Purview | ϕ |
| --- | --- | --- | --- | --- |
|  | (0) | (5) | (2) | 0.25 |
|  | **(1)** | **(5)** | **()** | **0** |
|  | **(2)** | **(0)** | **(3, 4)** | **0.167** |
|  | (3) | (2) | (5) | 0.25 |
|  | (4) | (2) | (5) | 0.25 |
|  | (5) | (3, 4) | (0, 1) | 0.167 |

We then consider a black-box system of two COPY elements over two time steps. The black-box elements are constituted of micro elements A = (0, 1, 2) and B = (3, 4, 5), with corresponding output elements 2 and 5. The cause-effect structure of the black-box system is:

| black box  unpartitioned | Mechanism | Past Purview | Future Purview | ϕ |
| --- | --- | --- | --- | --- |
|  | A | B | B | 0.5 |
|  | B | A | A | 0.5 |

The MIP for this system is to cut all connections from (0) to (1, 2, 3, 4, 5), that is, cut the outputs of micro element 0 (by symmetry, cutting the outputs of elements 1, 3 or 4 would be equivalent). After the partition, both mechanisms have had their irreducible cause-effect power diminished, and the result is Φ = 0.639.

| black box  partitioned | Mechanism | Past Purview | Future Purview | ϕ |
| --- | --- | --- | --- | --- |
|  | **A** | **B** | **B** | **0.167** |
|  | **B** | **A** | **A** | **0.25** |
