# Supplementary material for: Black-boxing and cause-effect power
Source: PLoS Comput Biol. 2018 Apr 23;14(4):e1006114. doi: 10.1371/journal.pcbi.1006114 (PMC5933815; doi:10.1371/journal.pcbi.1006114)
Supplement: S3 Text — Additional discussion on analyzing cause-effect power from the intrinsic perspective. (DOCX) [file pcbi.1006114.s003.docx]

**Black-boxing and cause-effect power:**

**Supplementary Information**

William Marshall^1^, Larissa Albantakis^1^, Giulio Tononi^1,^ *^*^*

^1^*Department of Psychiatry, Center for Sleep and Consciousness, University of Wisconsin, Madison, WI, USA*

*^*^Corresponding author: gtononi@wisc.edu*

S3 Text – The intrinsic perspective

The procedure for calculating the intrinsic cause-effect power of systems at a micro level is documented in detail in previous work (Oizumi et al., 2014, Mayner et al., 2016). Here we discuss aspects that deserve further comments when the analysis of intrinsic cause-effect power is applied to macro systems.

**Intrinsic cause-effect power**

To perform the intrinsic causal analysis of a system, its elements are perturbed with equal probability into all possible states and the resulting state transitions are observed. As with other interventional accounts of causality, by *setting* the elements into different states rather than merely observing them, true causal relationships can be distinguished from correlations. Additionally, perturbing the system into all possible states allows all counterfactuals to be evaluated, to assess the specificity of the causal relationship between elements and quantify cause-effect power. The goal of this exhaustive perturbational analysis is to unfold the full intrinsic cause-effect power of the system, that is, how the current state of system elements constrains their potential past and future states. Importantly, when analyzing the system at the micro level the micro elements of the system are set into all their possible micro states with equal probability. The same is done when analyzing the system at the macro level, except that this time it is the macro elements that are set into all their possible macro states with equal probability. In general, equal probability for macro states does *not* correspond to a uniform distribution of micro states.

When performing intrinsic causal analysis on a black-box system, some specific considerations have to be taken into account. Most importantly, one must capture the constraints due to the macro state of the black box itself (its output) but not any constraints due to the state of the micro elements within the black box. In this regard, it is useful to consider micro elements that fall into three different categories; micro elements that impose constraints within their corresponding macro element (Fig A-A), micro elements that impose constraints on other black boxes (Fig A-B; connection from F to D), and micro elements that are outside the system (Fig A-C; D is outside the system).

A single macro state may be consistent with multiple micro states of its micro elements, which nevertheless may impose different constraints on the past/future states of the system. Such micro constraints that do not originate from a black box’s output element at the relevant macro update must be discounted throughout the macro causal analysis and are treated as noise. For example, consider the black-box element γ in Fig. A-A, which has micro elements C and D as inputs (receiving from the output elements of black boxes α and β respectively) and E as its output. There is feedback within this black box (bidirectional connection between micro elements C and D), hence the state of micro elements C and D matters when specifying the input-output relationship of the black box (see Table 2). However, the state of constituent micro elements should not contribute to the cause-effect power of a macro system. Therefore, when perturbing the system into a particular macro state, the relevant micro elements are perturbed with equal probability into all possible micro states that are consistent with the macro state. In practice, this procedure amounts to averaging across the input-output relation for all possible states of the hidden micro elements within the black box, resulting in the truth table shown in Fig A-A, which is the average of the four truth tables resulting from the four possible states of CD (00, 10, 01, 11) (Table 2). Another instance (not dealt with in the current work) is when the output of a generalized macro element (black box) is defined as a coarse-graining of micro elements. In this case, the specific identity of these micro constituents should not contribute to the intrinsic cause-effect power of the macro system. However, in certain situations, such as when a single micro element from one coarse-grained macro element provides output to two different macro elements, constraints due to specific micro constituents can manifest in the macro TPM as “instantaneous causation” or “conditional dependence” (i.e., the current state of a macro element constrains the current state of other macro elements) and must be discounted in the causal analysis.

Another type of micro constraint that needs to be discounted while evaluating the intrinsic cause-effect power of a macro-level system is ‘lateral’ connections between black boxes, i.e. when a micro element hidden within a black box constrains a micro element within a different black box. To ensure that only constraints due to the state of macro elements are captured in the analysis, all connections originating from micro elements within a black box and terminating outside of it are injected with noise. This includes not only connections belonging to micro elements hidden within the black box, but also the micro output element of the black box at times other than the macro time step being considered. For example, in Fig. A-B, the connection from micro element F to micro element D must be noised, since F is not an output element of a black box. Similarly, when assessing whether black-box elements α and β constrain the future state of δ over 4 time steps, the output element E of γ must be noised throughout the macro update. This is to ensure that constraints due to the state of micro elements within γ are not counted towards the cause-effect power of α or β, which here do not constrain δ directly. The black-box element γ can be thought as “screening off” the indirect effect of α and β on δ, because α and β can only effect δ via the intermediate element γ.


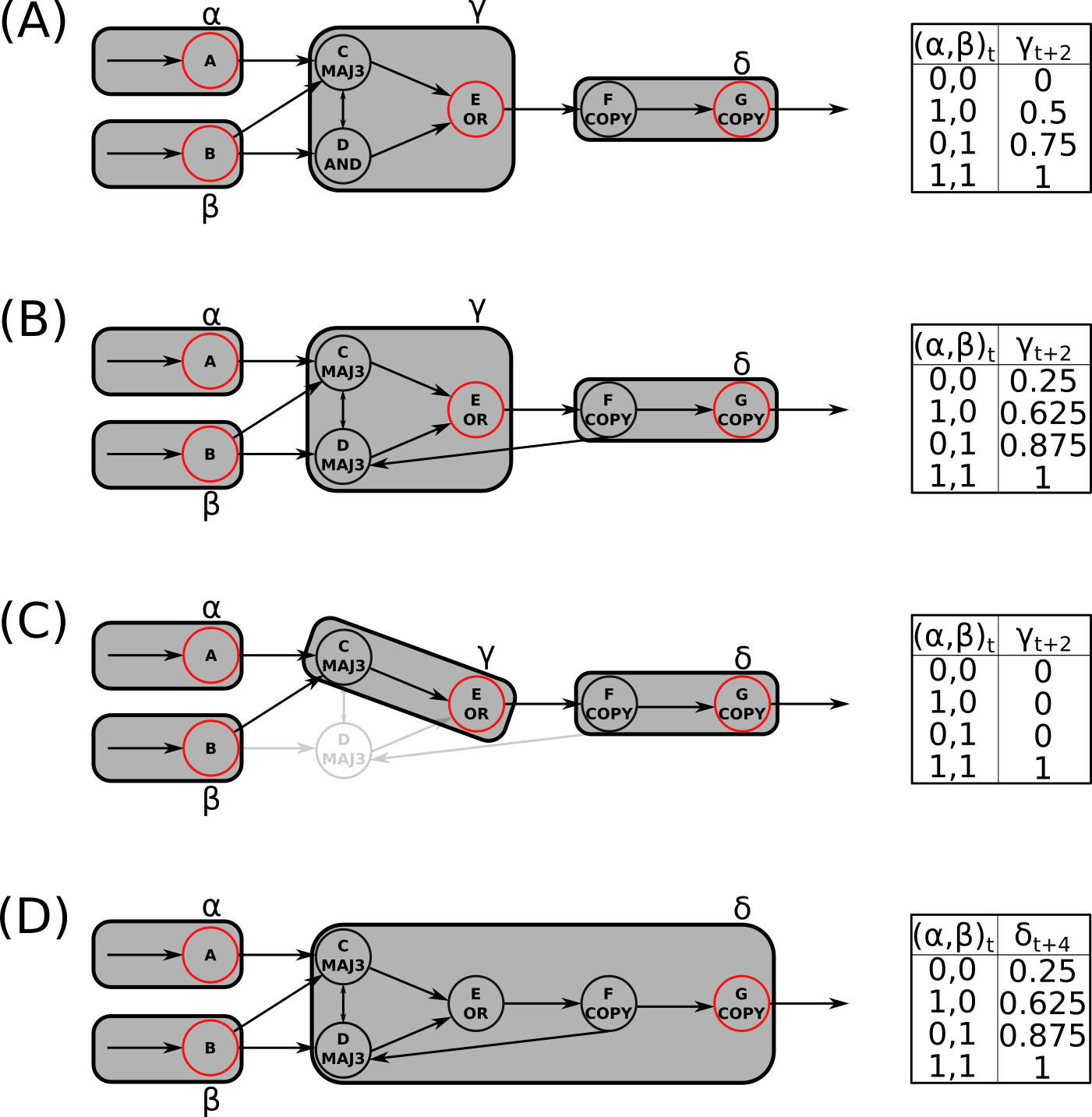


**Figure A**: Examples of partial systems of micro elements (A, B, C, D, E, F, G) with several different black-boxings into macro elements and the corresponding input-output function. (A): Four black-box elements α, β, γ, δ. Elements α and β have a joint constraint on γ, but it is not fully specific (there is indeterminism). All outputs of hidden micro elements are internal to their corresponding black box. (B): Similar to the (A) except constituent element F has an output that leaves its black box. The effect of F on D is not intrinsic to the macro system and must be noised during the entire causal analysis. As a result, the effects on gamma are less deterministic. (C): A similar black-boxing as in (B), except micro element D is outside the system, rather than within γ. The element D in its current state 0 is thus taken as a background condition. In this situation, the effect of α and β on γ is more specific. (D): A potential black-boxing with only three elements. In panels (A), (B) and (C), the effect of α and β on δ is screened-off by γ. In this case, the micro connection from E to F is within a black box rather than between so α and β have a direct effect on δ (it is no longer screened-off).

Table 2: Input-output relation for different initial states of hidden micro elements (C, D) of black box γ in panel (A) of Fig. A.

| If (C, D)_t_ = (0, 0) | | |  | If (C, D)_t_ = (1, 0) | | |
| --- | --- | --- | --- | --- | --- | --- |
| (A, B)_t_ | (C, D)_t+1_ | E_t+2_ |  | (A, B)_t_ | (C, D)_t+1_ | E_t+2_ |
| 0, 0  1, 0  0, 1  1, 1 | 0, 0  0, 0  0, 0  1, 0 | 0  0  0  1 |  | 0, 0  1, 0  0, 1  1, 1 | 0, 0  0, 0  0, 1  1, 1 | 0  0  1  1 |
|  | | |  |  | | |
| If (C, D)_t_ = (0, 1) | | |  | If (C, D)_t_ = (1, 1) | | |
| (A, B)_t_ | (C, D)_t+1_ | E_t+2_ |  | (A, B)_t_ | (C, D)_t+1_ | E_t+2_ |
| 0, 0  1, 0  0, 1  1, 1 | 0, 0  1, 0  1, 0  1, 0 | 0  1  1  1 |  | 0, 0  1, 0  0, 1  1, 1 | 0, 0  1, 0  1, 1  1, 1 | 0  1  1  1 |

In Fig. A-B, both micro elements C and D constrain the output element E, hence C and D would naturally seem to belong inside the black box γ. Nevertheless, the search for local maxima of intrinsic cause-effect power must consider all alternatives, including one in which D is taken to be a background condition rather than a hidden element within γ (or part of any other black box). In this alternate system (Fig. A-C), the state of D (OFF) is fixed as a background condition. In this case, the input-output relation for black box γ changes to the one shown in Fig A-C. From the figure alone, one cannot determine which of the two systems (top or middle panel) should qualify as the local maximum. However, it is apparent that the effect of α and β on γ is not fully deterministic when D is hidden inside γ, but it becomes deterministic when D is treated as a background condition. Furthermore, the repertoire of possible past states of γ is more degenerate when D is hidden inside γ as compared to when D is treated as a background condition (in this case there is only one possible past state of γ = 1, while with D inside the black box all four states of α and β could have led to γ = 1 with some probability). Thus, everything else being equal, the system with D as a background condition is more deterministic and less degenerate than the system with D hidden inside γ and should therefore have higher Φ. This result suggests that even if, from an extrinsic perspective, a set of micro elements may appear to constitute a macro element, from the intrinsic perspective only the set of micro elements that contribute to maximizing cause-effect power (i.e., the “skeleton” mediating the strongest constraints) actually constitutes the macro element.

**Integration and Exclusion**

When evaluating cause-effect power at macro scales, we have to consider the micro elements that *constitute* the macro-level system. As stated in the main text (section “Black-boxing” parts iii and iv), the integration and exclusion postulates both apply to the constituents of black-box systems. By the integration requirement, the set of constituents must be irreducible; two unrelated systems cannot be black-boxed together since their constituents are not integrated (Fig. B-A, B-B). Moreover, micro input (or output) elements cannot be black-boxed (Fig. B-C) because they lack causes (or effects) within the system. Only when the set of constituents is integrated (Fig. B-D) can a macro system be integrated.


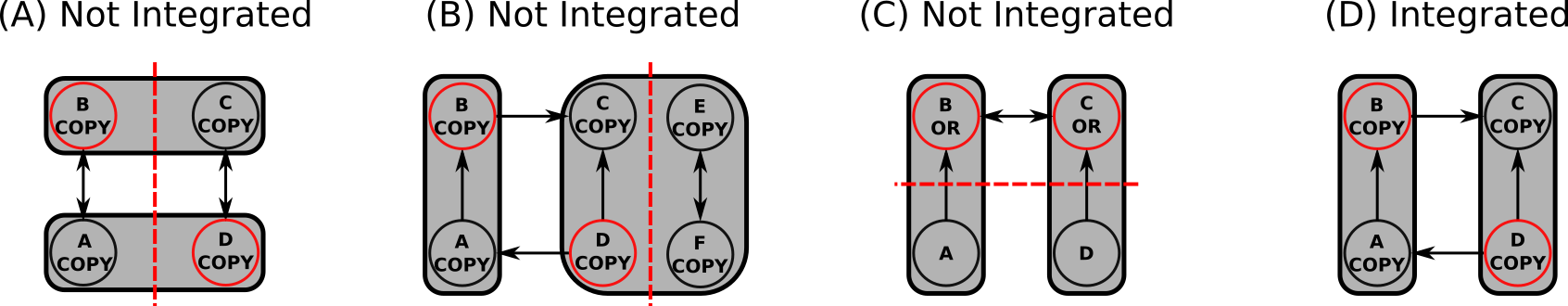


***Figure B****: Four examples of potential black-box systems and their constituent micro elements. The constituents of a macro system must be irreducible (integration). Among the examples in the figure only (D) has a properly integrated set of constituents, while (A), (B) and (C) are reducible, with the corresponding cut drawn as a dashed red line. (A, B) Two systems that are not integrated at the micro level cannot constitute a macro, black-boxed system; (C) Elements that provide only inputs to (or only outputs from) the system cannot be constituents of a black box.*

By the exclusion postulate, a (macro) element must be definite. Thus, a micro constituent cannot contribute to multiple black boxes within a system (Fig. C).


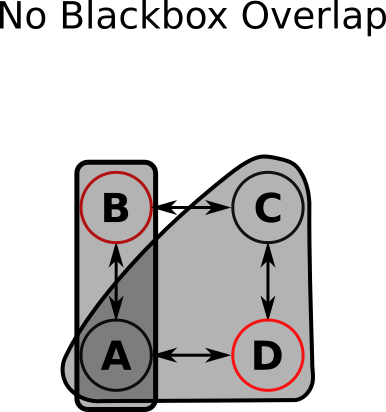


***Figure C****: Two examples of micro constituents having their cause-effect power double counted. (A) The micro element A is contributing to two different black-box elements (A, B) and (A, C, D). The exclusion postulate rules out this potential black-box system.*
